# Hot Deformation Behavior and Microstructural Evolution of a TiB_2_/Al-Zn-Mg-Cu-Zr Composite

**DOI:** 10.3390/ma17071487

**Published:** 2024-03-25

**Authors:** Jingcun Huang, Zhilei Xiang, Meng Li, Leizhe Li, Ziyong Chen

**Affiliations:** Faculty of Materials and Manufacturing, Beijing University of Technology, Beijing 100124, China; jchgrinm@hotmail.com (J.H.);

**Keywords:** composites, hot deformation, constitutive equation, microstructure

## Abstract

In the present work, the hot deformation behavior and microstructural evolution of a TiB_2_/Al-Zn-Mg-Cu-Zr composite were studied. Hot compression tests were conducted within a temperature range of 370 °C to 490 °C and a strain rate of 0.001 s^−1^ to 10 s^−1^. We established the Arrhenius constitutive equation with Zener–Hollomon parameters and processing maps and discussed the microstructural evolution during hot deformation. The results indicated that the safe processing parameter region falls within 370 °C–490 °C and 0.001 s^−1^–0.025 s^−1^. The influence of the strain rate on the safe processing range is more dominant than that of deformation temperature, which is primarily attributed to TiB_2_. Dynamic softening is primarily governed by dynamic recovery (DRV). Small particles (η, Al_3_Zr) can pin dislocations, promoting the rearrangement and annihilation of dislocations and facilitating DRV. Higher temperatures and lower strain rates facilitated dynamic recrystallization (DRX). Continuous dynamic recrystallization (CDRX) occurs near high-angle grain boundaries induced by strain-induced boundary migration (SIBM). TiB_2_ and large second-phase particles generate high-density geometrically necessary dislocations (GNBs) during hot deformation, which serve as nucleation sites for discontinuous dynamic recrystallization (DDRX). This enhances dynamic softening and improves formability.

## 1. Introduction

Alloys in the Al-Zn-Mg-Cu series are widely used in the automotive, aircraft, and aerospace industries due to their high strength–weight ratio, specific strength, good corrosion resistance, and excellent formability [1,2,3]. With the increasing demand for lightweight materials in various fields, the application of high-strength aluminum alloys is also rapidly expanding. In order to further break through the limits of the mechanical properties of aluminum alloys, aluminum matrix composites have become an important research direction [4,5]. TiB_2_ particles act as outstanding reinforcements due to the fact that they exhibit a high melting point, high hardness, high elastic modulus, good thermal stability, and good coherence with Al matrices [6,7,8,9]. Thus, TiB_2_ particles have been widely used as some of the most ideal reinforcements for Al-Zn-Mg-Cu matrix composites.

Hot processing is a commonly employed treatment for eliminating metallurgical defects, addressing the clustering of reinforced particles, modifying microstructures, and reconciling the conflict between strength and plasticity [10,11,12]. Identifying the optimal hot deformation processing parameters (such as the deformation temperature and strain rate) is essential for producing desirable microstructures that enhance the mechanical properties of a composite.

Understanding the hot deformation behavior of materials is essential for designing the hot processing parameters to achieve the best matching between the deformation microstructure and dynamic softening microstructure. In recent years, many studies have been carried out in the field of hot deformation and microstructural evolution for aluminum alloys. According to the Arrhenius model with the Zener–Hollomon parameters, semi-empirical and semi-theoretical constitutive equations were derived, and these equations predicted the flow stress well [13,14]. Mirzadeh [15] analyzed the hot deformation of the 7075 alloy to build a constitutive equation that described the changes in flow stress during hot deformation. Obtaining the optimal hot deformation processing parameters through processing maps is also a key direction in the research on the hot deformation of Al-Zn-Mg-Cu alloys [16,17,18]. For Al-Zn-Mg-Cu alloys, dynamic recovery (DRV) has been proven to be the dominant softening mechanism during hot deformation because of the high stacking fault energy [19,20,21]. At strain rates surpassing the critical threshold, dynamic recrystallization (DRX) manifests exclusively in proximity to the initial grain boundary. Conversely, below the critical strain rate, DRX transpires through grain fragmentation [22]. Nucleation during recrystallization is very complex in nature. The local thermal gradient plays a decisive role in nucleation [23,24,25]. Numerous studies [26,27,28,29,30,31] have revealed that the DRX mechanism is dominated by continuous dynamic recrystallization (CDRX) and discontinuous dynamic recrystallization (DDRX) in Al-Zn-Mg-Cu alloys. DDRX is the main mechanism, and the proportion of DDRX increases with increasing strain rate, decreasing temperature, and increasing deformation degree. Zhao et al. [30] investigated the hot deformation behavior and workability of the 7055 aluminum alloy. The recommended processing parameters were determined to be 300–400 °C and 10^−1.5^–1 s^−1^, and good workability and deformation microstructures could be obtained under these conditions. Tang et al. [31] suggested that a high Zn content in Al-Zn-Mg-Cu alloys can promote the dynamic softening during hot deformation by promoting dynamic precipitation. Raja et al. [32] reported a wider processing parameter range for Al-7.3Zn-2.2Mg-2Cu under 50% compression. A high recrystallization fraction (73%) microstructure including CDRX and particle-stimulated nucleation (PSN) was obtained at 450 °C and 0.001 s^−1^.

For aluminum matrix composites, reinforcement particles promoted the generation of geometrically necessary dislocations (GNDs) and reduced the mobility of GNDs, effectively meeting the energy requirements for DRX [33]. This further consolidated the dominant effect of DDRX induced by PSN. Reinforced particles are systematically categorized to analyze their impact on recrystallization or pinning effects [34]. The prevalent theory concerned with assessing the influence of reinforced particles on recrystallization is primarily based on their sizes. Particles larger than 1 μm activate the (PSN) mechanism, thereby enhancing the fraction of recrystallized microstructures [35,36]. Fine particles, conversely, impede or potentially suppress recrystallization [37,38]. The distribution of particles also results in various pinning effects on the grain boundary motion. Reinforcement particles exert a robust pinning influence on grain boundaries, effectively impeding the coarsening of recrystallized grains [39,40]. However, due to the introduction of reinforced particles (especially TiB_2_ particles), the complexity of the material system results in insufficient understanding of the hot deformation behavior and microstructural evolution of composites. Although much research work on the effect of reinforcement particles on microstructural evolution—especially on the dynamic recrystallization mechanism—has been carried out, there are few studies about reinforcement particles in the range of safe processing parameters, the evolution of the microstructure of deformations, and the mechanism of influence of unstable microstructures for TiB_2_-particle-reinforced Al-Zn-Mg-Cu-Zr composites.

In the present study, the hot deformation behavior of a 6 wt.% TiB_2_/Al-Zn-Mg-Cu-Zr composite that was prepared in situ was investigated with a hot compression test. The constitutive equations are established, and the microstructural evolution and dynamic softening mechanisms are discussed in detail.

## 2. Materials and Methods

According to previous research, 6 wt.% TiB_2_-reinforced Al-Zn-Mg-Cu-Zr composite has good comprehensive mechanical properties, including high strength, stiffness, and acceptable plasticity [41]. Thus, in this study, a 6 wt.% TiB_2_/Al-Zn-Mg-Cu-Zr composite was investigated; the composition of the elements is listed in Table 1. The raw materials included pure commercial Al, Zn, Mg (purity 99.5%), Al-(50 wt.%) Cu, Al-(4 wt.%) Zr, and Al-(20 wt.%) TiB_2_. The Al-(20 wt.%) TiB_2_ master alloy was prepared using the in situ self-propagating high-temperature synthesis (SHS) method, resulting in TiB_2_ particles with a size range of 0.2–3 μm [42]. The casting processing may have caused the loss of a small number of TiB_2_ particles. The as-cast ingot underwent homogenization annealing (400 °C/4 h + 465 °C/24 h) followed by gradual cooling in ambient air. The as-homogenized TiB_2_/Al-Zn-Mg-Cu-Zr composite was then cut into a Ф10 mm × 15 mm cylindrical specimen for hot compression testing on a Gleeble-3500 at different temperatures (370 °C, 400 °C, 430 °C, 460 °C, 490 °C) and strain rates (0.001 s^−1^, 0.01 s^−1^, 0.1 s^−1^, 1 s^−1^, 10 s^−1^) under vacuum. The tested specimens underwent mist cooling for quenching and mid-plane sectioning along the compression axis with prepared cut sections for microstructure examination. The central region was chosen for observing microstructures.

Scanning electron microscopy (SEM) analysis was performed using a QUANTA FEG 650 (FEI, Hillsboro, OR, USA) equipped with an energy-dispersive X-ray spectrometer (EDS, Oxford X-MAXN-80, Oxford Instruments, Abingdon, UK) and electron backscattered diffraction (EBSD) detector at 20 kV. The working distance and scanning step in EBSD testing were 15 mm and 3 μm. EBSD samples were extracted from the middle of hot compression samples and electrolytically polished (at 20 V and 0.6 A for 15 s) using a 10% perchloric acid and 90% methanol mixture at room temperature. The EBSD data were processed using Channel 5 with a moderate degree of cleaning of unresolved areas. Microstructures, including recrystallization, substructures, and deformed microstructures, were identified according to the average misorientation value: recrystallized microstructure, average misorientation angle of <2°; substructure, average misorientation angle of 2°–7.5°; deformed microstructure, average misorientation angle of >7.5°. This was because recrystallized grains continue to generate an internal orientation gradient during the continuous hot deformation process. Thus, some manual methods were used to select REXed grains as follows: 1. average grain misorientation angle of less than 2°; 2. grains with over 50% high-angle grain boundaries; 3. single small grains with an equiaxed shape and high-angle grain boundaries. Transmission electron microscopy (TEM) analysis was carried out on an FEI Talos F200X-G2 microscope (Hillsboro, OR, USA) operated at an accelerating voltage of 200 kV. The TEM samples were prepared by mechanically thinning φ3 mm × 100 μm disks. Thinned samples were further subjected to twin-jet polishing using a solution (30% nitric acid and 70% alcohol).

## 3. Results and Discussion

### 3.1. Plastic Flow Behavior in Hot Compression

**Figure 1:**
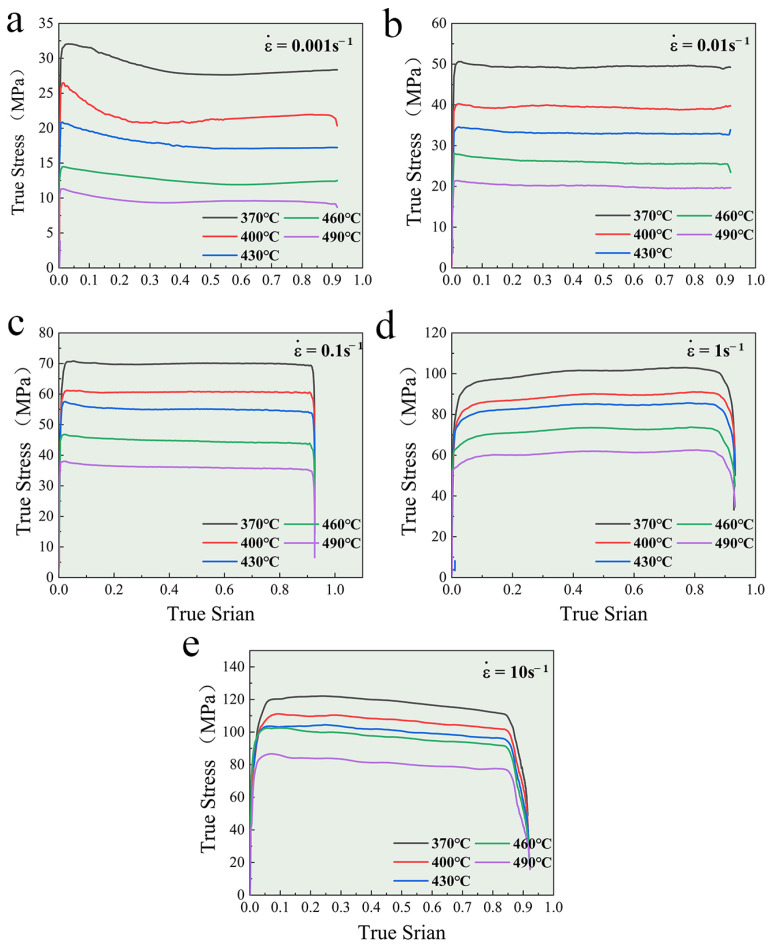
True stress–strain curves of the TiB_2_/Al-Zn-Mg-Cu-Zr composite. (**a**) $\dot{\varepsilon }=0.001\;s^{-1}$, (**b**) $\dot{\varepsilon }=0.01\;s^{-1}$, (**c**) $\dot{\varepsilon }=0.1\;s^{-1}$, (**d**) $\dot{\varepsilon }=1\;s^{-1}$, (**e**) $\dot{\varepsilon }=10\;s^{-1}$.

The flow stress–strain curves for the 6 wt.% TiB_2_/Al-Zn-Mg-Cu-Zr composite under various hot compression conditions are presented in Figure 1. Initially, rapid work hardening leads to the peak strain at a low true stain. Higher strain rates accelerate dislocation multiplication and energy storage and reduce the dynamic softening time. The peak true stress increases with the strain rate, which ranges from 0.001 s^−1^ to 10 s^−1^. The deformation temperature drives dislocation slipping and sub-boundary or grain boundary migration. Consequently, the peak true stress decreases with the increase in deformation temperature, which ranges from 370 °C to 490 °C. Work hardening is attributed to the increased dislocation density induced by deformation. With ongoing deformation, the composite’s microstructure tends towards a stable state through the dynamic softening mechanism to release stress. Upon reaching the peak true stress, the deformation behavior achieves a dynamic balance between work hardening and dynamic softening. Dynamic softening encompasses DRV and DRX [43]. During the uniform deformation process, the true stress slightly decreases under low-strain-rate conditions. This indicates the dominant role of the dynamic softening mechanism at low strain rates and the restriction at high strain rates. Additionally, at 0.001 s^−1^, elevated deformation temperature enhances thermal activation [44,45], reduces the energy barrier of dislocation slipping, and releases deformation energy storage over time, causing almost no decrease in true stress.

### 3.2. Constitutive Equation

Hot processing of composite a is a thermal activation process. Sellars and Tegart [46] proposed an empirical formula to calculate and describe the relationships between the strain rate, deformation temperature, and flow stress during the thermal deformation of materials. It was found that these factors satisfy Arrhenius-type equations and can be expressed in the form of a hyperbolic sine. At the same time, a modified relationship including the deformation activation energy and deformation temperature was proposed, and it is widely used to describe the deformation behavior of materials with thermal activation [47,48]. The relations between the true stress, temperature, and strain rate are described by Equations (1)–(3). Equation (1) can describe the hot flow behavior for the whole stress range well, and Equations (2) and (3) apply to the low-flow-stress range (ασ < 0.8) and high-flow-stress range (ασ > 1.2), respectively [47,48].

(1) $$\dot{\varepsilon }=A{\left[\sin h\left(\alpha \sigma \right)\right]}^{n}\exp\left(-Q/RT\right)$$

(2) $$\dot{\varepsilon }=A_{1}\exp\left(\beta \alpha \right)\exp\left(-Q/RT\right)$$

(3) $$\dot{\varepsilon }=A_{2}\sigma^{n_{1}}\exp\left(-Q/RT\right)$$

where $\dot{\varepsilon }$ is the strain rate (s^−1^); *A*, *A*_1_, and *A*_2_ are the structure factors; α represents the coefficient of material stress (MPa^−1^); *σ* is the true stress (MPa); *n* and *n*_1_ are stress indexes; *R* is the gas constant; *Q* and *T* are the deformation activation energy (kJ/mol) and deformation temperature (K), respectively. Additionally, *β* is a constant, and the relation among *β*, *α*, and n1 is *α* = *β*/*n*_1._

The Zener–Hollomon parameter can be employed to predict hot deformation behavior, and the equation is expressed as follows [47]:

(4) $$Z=\dot{\varepsilon }\exp\left(Q/RT\right)$$

To further deduce the equations, natural logarithms are taken on both sides of Equations (1)–(4), and the corresponding formulas are listed as follows (Equations (5)–(9)):

(5) $$\ln\dot{\varepsilon }=\ln A+n\ln[\sin h(\alpha \sigma )]-Q/RT$$

(6) $$\ln\dot{\varepsilon }=\ln A_{1}+\beta \sigma -Q/RT$$

(7) $$\ln\dot{\varepsilon }=\ln A_{2}+n_{1}\ln\sigma -Q/RT$$

(8) $$\ln Z=\ln\dot{\varepsilon }+Q/RT$$

(9) $$\ln Z=\ln A+n\ln[\sin h\left(\alpha \sigma \right)]$$

**Figure 2:**
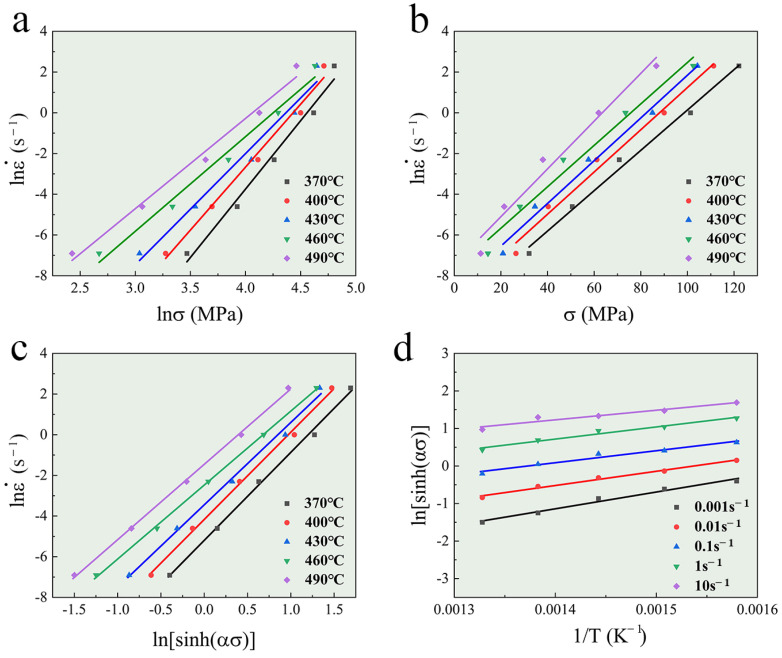
Linear relationships of (**a**) lnσ-ln, (**b**) σ-ln$\dot{\varepsilon }$, (**c**) ln[sinh(ασ)]-ln$\dot{\varepsilon }$, and (**d**) T-ln[sinh(ασ)].

**Figure 3:**
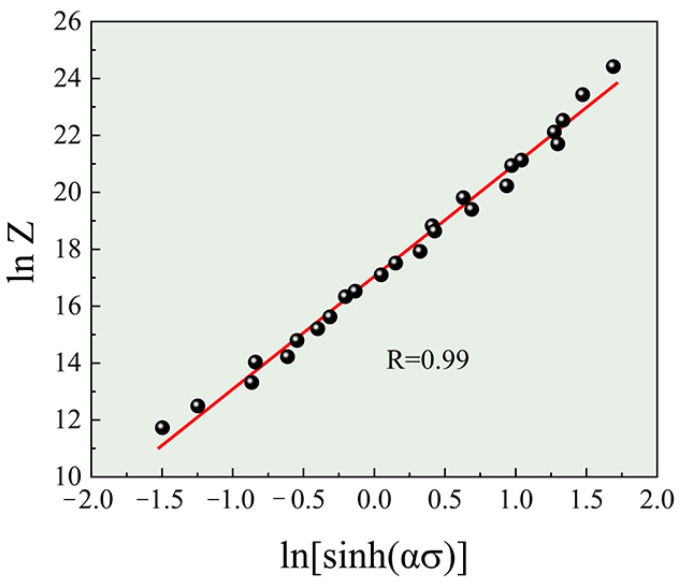
Linear relationship between lnZ and ln[sinh(ασ)].

Based on the data from the hot compression experiments, the linear fit plots of lnσ-$\ln\dot{\varepsilon }$, σ-$\ln\dot{\varepsilon }$, ln[sinh(ασ)]-$\ln\dot{\varepsilon }$, and 1/T-ln[sinh(ασ)] are shown in Figure 2. Table 2 presents the average values of the parameters calculated from the slopes and intercepts. The deformation activation energy (*Q*) reflects the energy barrier for dislocation movement during hot deformation. According to the linear relationship of 1/T-ln[sinh(ασ)], the *Q* value can be calculated as 118.26 kJ. To describe the hot deformation behavior in all of the conditions, the value of *Q* is inserted into Equation (9) to calculate the constitutive equation containing the Zener–Hollomon parameter. Figure 3 illustrates the linear fit plot of lnZ-ln[sinh(ασ)]. The values of n and A can be obtained with the slope and intercept, and they are 3.96 and 2.53 × 10^7^, respectively. Substituting all the constant values into the given formulas, the constitutive equations for the TiB_2_/Al-Zn-Mg-Cu-Zr composite are as follows (Equations (10) and (11)):

(10) $$\dot{\varepsilon }=2.53\times 10^{7}{\left[\sin h\left(0.023\sigma \right)\right]}^{3.96}\exp\left(-118.26/RT\right)$$

(11) $$\sigma =44.25\ln\left\{{\left(\frac{Z}{2.53\times 10^{7}}\right)}^{\frac{1}{3.96}}+{\left[{\left(\frac{Z}{2.53\times 10^{7}}\right)}^{\frac{2}{3.96}}+1\right]}^{0.5}\right\}$$

### 3.3. Processing Maps

In accordance with dynamic material modeling (DMM) [49], hot deformation is considered a process of power dissipation. Under a given strain condition, the power dissipation efficiency (η) varies with the deformation temperature and strain rate, forming a power dissipation map where different regions may correspond to specific microstructural mechanisms. Typically, regions with higher power dissipation efficiency correspond to better machinability. The efficiency of power dissipation can be expressed as Equation (12) [50]:

(12) $$\eta =\frac{2m}{m+1}$$

where m is the strain rate sensitivity, and it can be expressed as Equation (13).

(13) $$m={\left[\frac{\partial \left(\ln\sigma \right)}{\partial (\ln\dot{\varepsilon })}\right]}_{\varepsilon ,T}$$

Following the principle of the maximum entropy generation rate, the criterion for the instability parameter (ξ) can be described as Equation (14) [51,52]. If ξ < 0, there is a high likelihood of adiabatic shear bands, microscopic pores, cracks, and triangular grain boundary cracking occurring inside the material.

(14) $$\xi =\frac{\partial \ln(\frac{m}{m+1})}{\partial \ln\dot{\varepsilon }}+m$$

**Figure 4:**
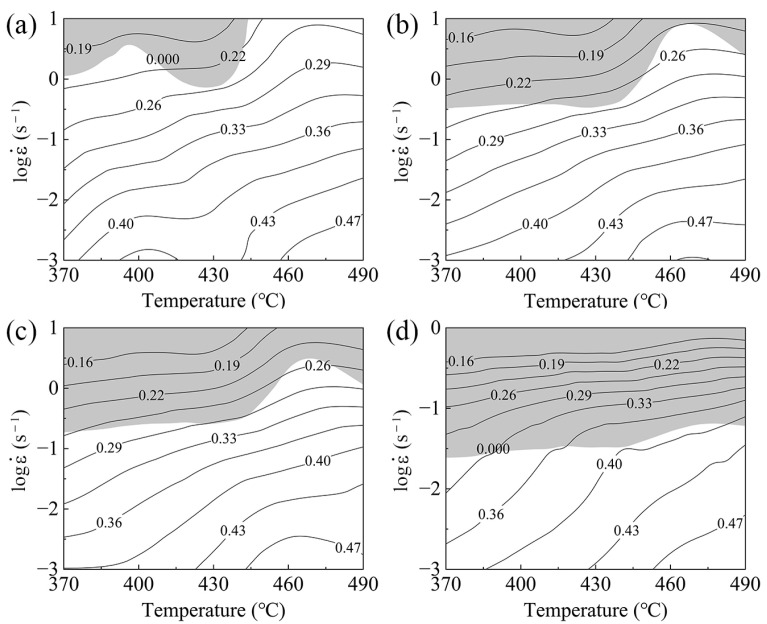
Processing maps of the TiB_2_/Al-Zn-Mg-Cu-Zr composite at true strains of (**a**) 0.3, (**b**) 0.5, (**c**) 0.7, and (**d**) 0.9.

Figure 4 displays the processing maps at true strains (0.3, 0.5, 0.7, and 0.9) for the TiB_2_/Al-Zn-Mg-Cu-Zr composite. Gray regions signify instability, while the white area denotes microstructural stability during hot deformation. The machinability range in the hot processing maps significantly shifts with the increase in the true strain. The stable processing range gradually narrows at low strain rates, with no notable change in the axis of the deformation temperature. Therefore, the safe processing area for the TiB_2_/Al-Zn-Mg-Cu composite is more responsive to variations in the strain rate than to the deformation temperature. The processing map indicates that at a strain of 0.9, the safe hot processing parameter ranges are 370–490 °C and 0.001–0.025 s^−1^. Compared with the matrix alloy (Al-Zn-Mg-Cu), reinforced particles (TiB_2_) play an important role in the hot processing parameters. Xu et al. [28] found that the optimal hot deformation conditions are 400–500 °C and 0.01~0.32 s^−1^ at the true strain of 1.2. Zhang et al. [53] established a hot processing map with a strain value of 0.6 for an Al-Zn-Mg-Cu alloy, which only indicated very small unstable processing areas, namely, 350–450 °C and 0.001–1 s^−1^. Under similar strain conditions, TiB_2_/Al-Zn-Mg-Cu composite materials have significantly more stringent hot processing ranges than those of matrix alloys (Al-Zn-Mg-Cu). This likely stems from a unique hot deformation phenomenon resulting from the introduction of TiB_2_ particles. The rapid geometrically necessary dislocation proliferation and dynamic recovery behavior induced by TiB_2_ particles are highly sensitive to changes in the strain rate. This increases the likelihood of locally uneven deformation due to dislocation interactions under high strain rates, causing instability in the material. The introduction of TiB_2_ particles greatly influenced the formation and migration of GNDs/boundaries, resulting in different main dynamic softening mechanisms and microstructural evolution. Further research focusing on diverse deformation temperatures and strain rates that inevitably lead to distinct microstructural evolution for the Al-Zn-Mg-Cu composite reinforced with TiB_2_ particles is described below.

### 3.4. Microstructural Evolution

**Figure 5:**
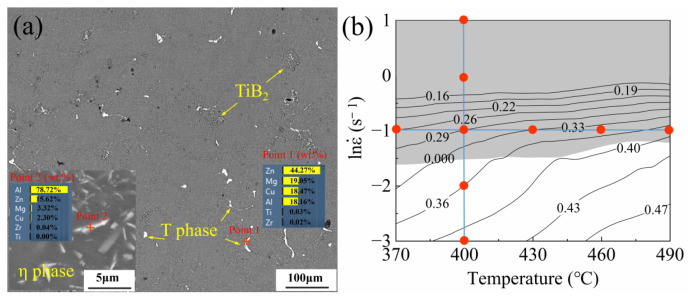
(**a**) SEM image of the original microstructure of the TiB_2_/Al-Zn-Mg-Cu-Zr composite (as-homogenized). (**b**) Processing map at a strain of 0.9; red points indicate samples under different deformation conditions for microstructural observations.

Figure 5 shows the initial microstructures of the hot deformation samples. The T phase, a common non-equilibrium eutectic phase for Al-Zn-Mg-Cu alloys, is composed of Al, Zn, Mg, and Cu (the EDS results were shown at Point 1). After homogenization, most of the T phase dissolved into the matrix, but a small amount the T phase remained in the microstructure. According to some traces, the locations of some original grain boundaries are outlined in Figure 5a. The TiB_2_ particles remained stable during homogenization, and they were distributed within the grains and at the grain boundaries. The inset shows an enlarged image of the η (Mg(Zn,Cu)_2_) phase, which precipitated from the matrix (supersaturated solid solution) during the gradual cooling to room temperature after homogenization. The η phase was mainly uniformly precipitated in the grains, and its morphology and contrast were obviously different from those of the T phase. The EDS results (Point 2) showed that the η phase was mainly composed of Mg and Zn, and the content of Cu was very small, which also made it very different from the T phase. EBSD was employed to further elucidate the microstructural evolution during hot deformation. Our investigation included an examination of the microstructural evolution at various strain rates and deformation temperatures (see Figure 5b), along with an assessment of the impact of TiB_2_ on the material instability and microstructural evolution during hot deformation.

**Figure 6:**
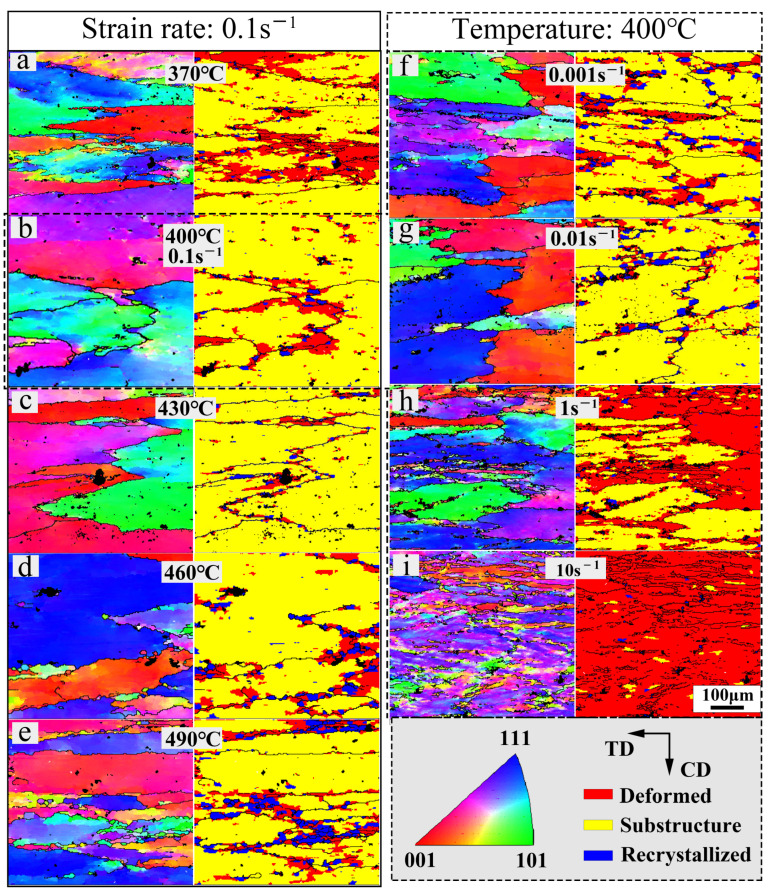
EBSD maps and grain orientation spread maps for different samples with various deformation temperatures and strain rates: (**a**) 370 °C/0.1 s^−1^, (**b**) 400 °C/0.1 s^−1^, (**c**) 430 °C/0.1 s^−1^, (**d**) 460 °C/0.1 s^−1^, (**e**) 490 °C/0.1 s^−1^, (**f**) 400 °C/0.001 s^−1^, (**g**) 400 °C/0.01 s^−1^, (**h**) 400 °C/1 s^−1^, and (**i**) 400 °C/10 s^−1^. (The blue region indicates recrystallized microstructures; the yellow region indicates substructures; the red region indicates deformed microstructures).

Figure 6 displays the inverse pole figures (IPFs) and grain orientation spread maps under various deformation conditions. Figure 6a–e show samples under deformation conditions situated at the edge of the instability region in the processing map (strain = 0.9). At 370 °C (Figure 6a), a small number of deformation shear bands were observed. A significant orientation gradient was present within the grains. With increasing temperature, the shear bands diminished, and the orientation gradient within the grains decreased. The grain orientation spread maps in Figure 6a–e show the microstructural evolution with rising deformation temperature. The dominance of substructures (yellow areas) signified that DRV was the primary dynamic softening mechanism. The fractions of substructures and dynamic recrystallization (blue areas) increased, while the fraction of deformed structures (red areas) decreased. Deformed structures (red areas) were initially distributed within the original grains at low deformation temperatures. With increasing temperature, they concentrated gradually towards the grain boundaries. Deformation temperature promoted DRX and the growth of DRXed grains. DRXed grains formed via both PSN and SIBM mechanisms are observed in Figure 6a–e. SIBM predominated at high deformation temperatures.

**Figure 7:**
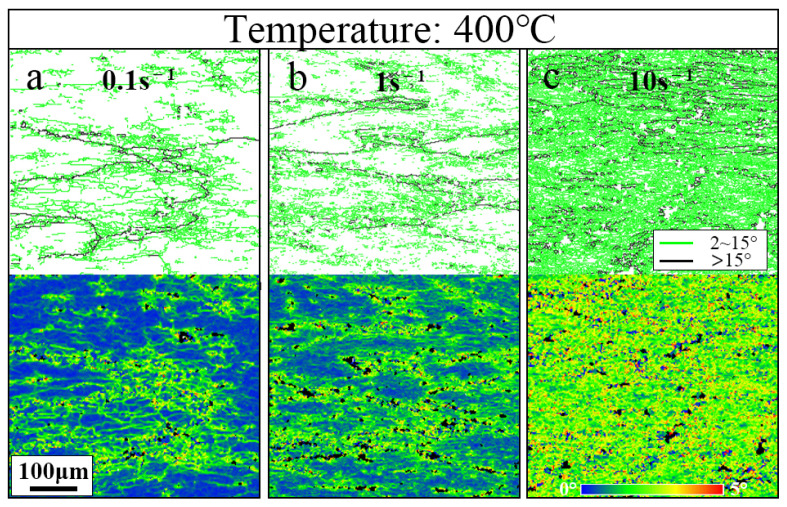
Grain boundary maps and KAM maps for different samples with various deformation strain rates: (**a**) 400 °C/0.1 s^−1^, (**b**) 400 °C/1 s^−1^, and (**c**) 400 °C/10 s^−1^.

Figure 6b,f–i display the IPF maps and grain orientation spread maps at various strain rates. Substructures remained dominant between 0.001 s^−1^ and 0.1 s^−1^, while deformed structures sharply increased at high strain rates (1 s^−1^, 10 s^−1^). The DRX fraction remained low at strain rates higher than 0.1 s^−1^ and increased with decreasing strain rates. Clearly visible deformation shear bands and significant uneven deformations were observed in samples subjected to high strain rates (Figure 6h,i), confirming the information from the hot processing maps. Elevated strain rates induced material instability, resulting in numerous microstructural defects. Further observation revealed that deformation shear bands were more likely to form in areas with a dense distribution of original grain boundaries and TiB_2_ particles. Figure 7 illustrates changes in the grain boundary distribution and kernel average misorientation (KAM) influenced by the strain rate (0.1 s^−1^, 1 s^−1^, 10 s^−1^). The low-angle grain boundary (LAGB), which is identified by the light green line, encompassed angles from 2° to 15°, while the high-angle grain boundary (HAGB) (misorientation angle greater than 15°) is marked by the black line. The KAM maps reflect the local disorientation of the composite and indirectly indicate the distribution of GNDs. Figure 7 shows the high density of low-angle grain boundaries (LAGBs) and KAM values near the original grain boundaries and TiB_2_ particles (black area in the KAM maps). As the strain rate increased, the density of LAGBs and KAM values significantly increased. During hot deformation, a high density of GNDs formed near the original grain boundaries and TiB_2_ in order to compensate for the difference in the deformation of the adjacent grains and the matrix–TiB_2_. Additionally, the hindering effect of the grain boundary and TiB_2_ led to a significant accumulation of dislocations here. High-rate deformation restricted dislocation slipping. In a short period, the rate of GND proliferation exceeded the migration rate, leading to rapid entanglement and hindrance of slipping. The accumulation of a large number of dislocations led to microstructural instability, resulting in local defects, such as deformation shear bands. However, at lower strain rates, dislocations had sufficient time to move to the grain boundary. Thus, DRV was promoted at low strain rates.

### 3.5. Dynamic Softening Mechanism

#### 3.5.1. Dynamic Recovery

**Figure 8:**
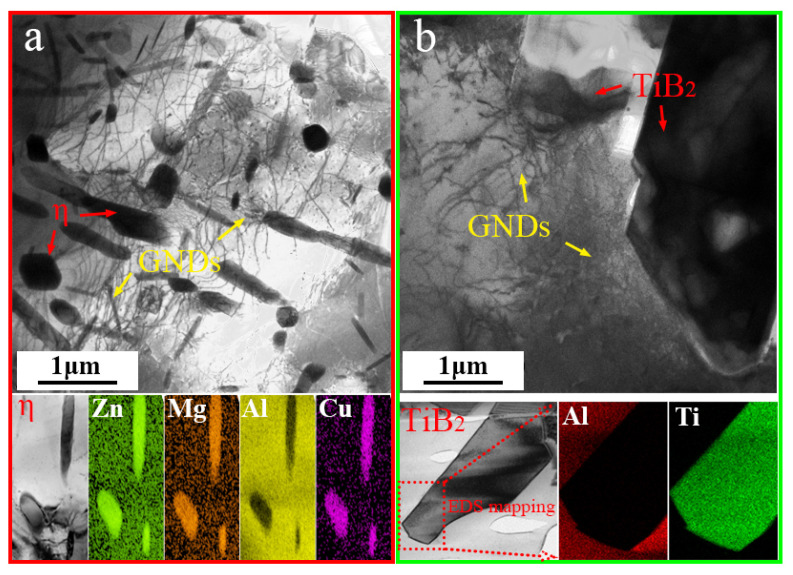
Effects of the second phases on the microstructure: (**a**) η phase; (**b**) TiB_2_ particles.

In the TiB_2_/Al-Zn-Mg-Cu-Zr composite, the deformation behavior of the FCC matrix with high stacking fault energy was facilitated by dislocation slipping. In the initial stage of hot deformation, the orientations of different primitive grains varied, as did most of the slippable directions of dislocations, resulting in an inhomogeneous distribution of internal stresses during deformation. As depicted in Figure 8, a huge difference in the elastic modulus between the TiB_2_ particles/residual T phase (large second phases) and the matrix resulted in a certain deformation gradient. To compensate for these differences, a large number of GNDs were generated. Additionally, the η phase (small second phases) can also produce this effect to a certain extent. As the strain continued, the dislocation density gradually increased, and when the internal energy storage of the material reached a critical value, dislocations started to slip. It is obvious that for the TiB_2_/Al-Zn-Mg-Cu-Zr composites, the TiB_2_ particles introduced a significant number of GNDs during the hot deformation process. The substantial difference in deformation ability compared to the Al matrix made the hot deformation behavior of the composite more sensitive to the strain and strain rate.

Second-phase (SP) particles have a significant impact on DRV. Prior to hot deformation, homogenization annealing was applied to the TiB_2_/Al-Zn-Mg-Cu-Zr composite. However, a small number of second-phase particles remained after annealing. Random trapping between dislocations and the pinning of small second-phase particles hindered the high-density dislocation slip driven by strain.

**Figure 9:**
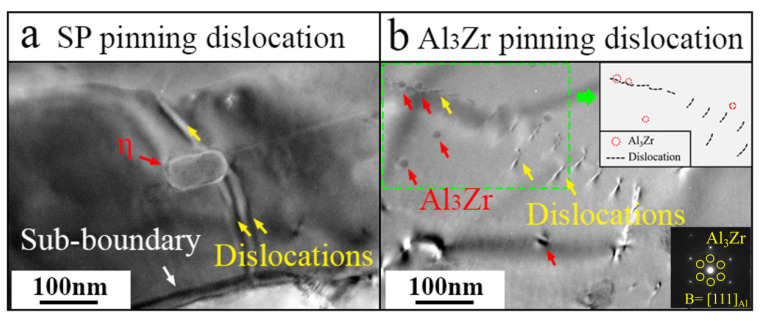
The interaction between the second-phase particles and dislocations: (**a**) η phase; (**b**) Al_3_Zr phase.

**Figure 10:**
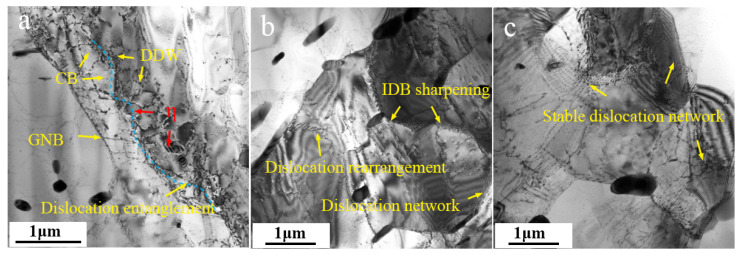
Dislocation structure and second-phase particle distribution in DRVed microstructures. (**a**) dislocation cell blocks, (**b**) sub-boundary forimg (**c**) stable dislocation network.

In Figure 9a, the small, short, rod-shaped SP particle might be the η phase. It pins dislocations inside a sub-grain. The pinning effect occurs when moving dislocations encounter SP particles. When the pinning effect is strong enough, the number of pinned dislocations gradually increases, and dislocation rearrangements and annihilation occur, forming a regular dislocation wall. The pinning effect of smaller particles is greatly reduced, and Figure 9b shows the pinning of Al_3_Zr particles on dislocations marked by the red arrows. For clarity, we have outlined the relationship between the position of the particle and the dislocation in the inset. Al_3_Zr particles are believed to precipitate during homogenization annealing, and their size is about 20 nm. Due to the weak pinning effect, small-sized particles induce a dislocation bypass mechanism, leaving a dislocation loop. Figure 10a shows the evolution of dislocations that are strongly pinned by large SP particles. As the deformation continues, tangled dislocations gradually evolve into dislocation clusters with a certain ordered arrangement. Driven by the deformation energy, dislocation clusters gradually connect to each other and form closed dislocation cell blocks (CBs). Then, as the dislocation density inside the CBs decreases further and that on the CB wall increases, the difference in orientation between CBs gradually increases. This CB structure is a relaxation structure aimed at reducing the overall energy during plastic deformation and internal energy storage, which is typical of the dynamic recovery process. Figure 10b,c suggest a pinning effect of SPs for dislocations. The CB wall pinned by SPs has a tendency to transform into a sharpening dislocation boundary with continuous deformation. During DRV, there are fewer dislocations inside CBs, cell wall dislocations are rearranged and eliminated, the CB wall is thinned and gradually sharpened, and stable dislocation networks that evolve into sub-boundaries are formed. Finally, if there is sufficient temperature and time, these sub-boundaries undergo overall pinning, further migration, and merging to form HAGBs. The internal orientation of the sub-grain will gradually rotate and tend to be consistent, forming DRXed grains.

#### 3.5.2. Dynamic Recrystallization

**Figure 11:**
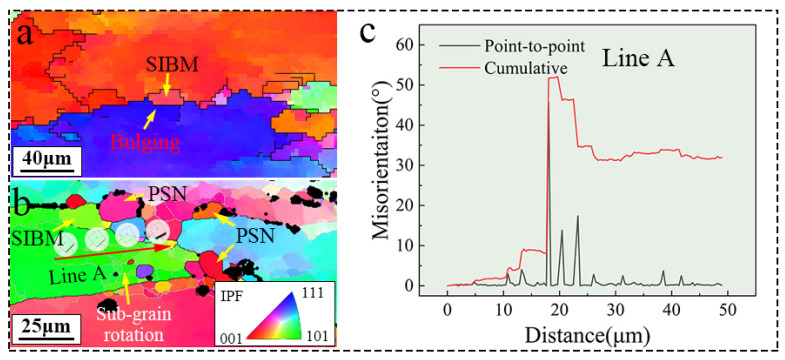
Structural characteristics for HAGB: (**a**) 490 °C/0.1 s^−1^; (**b**) 400 °C/0.1 s^−1^; (**c**) misorientation changes along Line A of panel (**b**).

DRXed grains formed with both the PSN and SIBM mechanisms were observed. Figure 11 shows a DRXed grain formed at the HAGBs induced by SIBM. In Figure 11a, the IPF maps reveal that the necklace-like DRXed grains had a similar orientation to that of the original grains at a higher deformation temperature. Moreover, the noticeable bulging of the grain boundary indicates that the necklace DRXed grains were dominated by CDRX. The SIBM mechanism easily occurred at high temperatures due to the activated migration of the original grain boundary induced by the strain and temperature. As shown in Figure 11b, within the triple-junction region, PSN, SIBM, and sub-grain rotation were present. The original triple junction is an easily clustered region for TiB_2_ or the T phase during composite solidification. Thus, PSN is the most important DRX mechanism. Additionally, based on the solidification characteristics of the TiB_2_/Al-Zn-Mg-Cu composite, the TiB_2_ particles tended to segregate at the original grain boundaries, which led to more complex dislocation structures at the original HAGBs, resulting in more complex dynamic recrystallization mechanisms there. The triple junction experienced higher stress concentration during hot deformation than that of Line-HAGB (Figure 11a), resulting in a greater driving force for the bulging of the original grain boundary. Therefore, a small amount of CDRX (SIBM) was also discovered, despite it being at a lower deformation temperature (400 °C). Moreover, Figure 11c shows that the point-to-point change in misorientation along Line A highlights the sub-boundaries with misorientations of less than 15°. The cumulative change line reveals the rotation of the sub-grains. Under stress, the DRV mechanism leads to the formation of sub-grains. Substructures then start to rotate, eventually transforming LAGBs into HAGBs.

**Figure 12:**
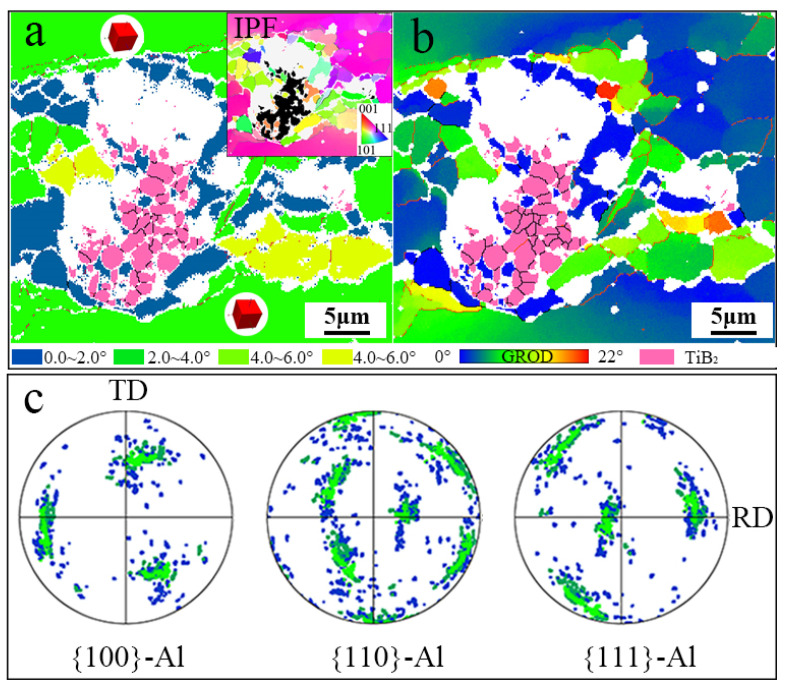
Structure around a TiB_2_ cluster (400 °C/0.1 s^−1^): (**a**) grain orientation spread map, and the inset is an IPF map; (**b**) GROD map; (**c**) PF maps for {100}-Al, {110}-Al, and {111}-Al.

In the TiB_2_/Al-Zn-Mg-Cu-Zr composite, reinforcement particles played a crucial role in DRX. The microstructure around the TiB_2_ particles is depicted in Figure 12. To distinguish them from other information, TiB_2_ particles are highlighted in pink in Figure 12a and Figure 12b, respectively. Due to shedding during mechanical and electrolytic polishing, TiB_2_ particles may have been present in the white unresolved region. Figure 12a is the grain orientation spread (GOS) map, considering that the GOS was less than 2°, as with the DRXed grains. However, the actual number of DRXed grains was likely larger than that marked in the GOS map because the initial DRXed grains continued to participate in deformation under sustained strain, causing an increase in the GOS value. The IPF map reveals a significant difference in orientation between the DRXed grains and the original grains. This suggests that the DRX around TiB_2_ was formed by DDRX and can be attributed to the PSN mechanism. The GROD map (Figure 12b) shows almost no residual stress in the DDRXed grains, and the GOS value is close to 0°. Nevertheless, a significant number of substructures were clustered in the outer layers of the DDRXed grains, with strong stress aggregation in the inner parts. These sub-grains continued to rotate with the strain until mature recrystallized grains were formed. The pole figures of {100}, {110}, and {111} are plotted in Figure 12c according to the GOS map (Figure 12a). The blue and green points represent DRXed and unDRXed grains, respectively. It is evident that the DDRXed grains with a random orientation weakened the texture of the deformed microstructure. Due to the introduction of TiB_2_ particles, DDRX was promoted during hot deformation.

## 4. Conclusions

1. The flow stress of the 6 wt.% TiB_2_-reinforced Al-Zn-Mg-Cu-Zr composite decreases with increasing deformation temperature and decreasing strain rate. The constitutive equations are expressed as
  $$\dot{\varepsilon }=2.53\times 10^{7}{\left[\sin h\left(0.023\sigma \right)\right]}^{3.96}\exp\left(-118.26/RT\right)$$
  $$\;\;\;\;\sigma =44.25\ln\left\{{\left(\frac{Z}{2.53\times 10^{7}}\right)}^{\frac{1}{3.96}}+{\left[{\left(\frac{Z}{2.53\times 10^{7}}\right)}^{\frac{2}{3.96}}+1\right]}^{0.5}\right\}$$

2. Processing maps were constructed, and the region of safe processing fell between 370 °C and 490 °C and 0.001 s^−1^ and 0.025 s^−1^. The strain rate exerts a more dominant influence on the safe processing range than that of the deformation temperature, which is primarily attributed to TiB_2_. The evaluated strain rate induces material instability, resulting in defects, such as high-strain stress and deformation shear bands within the composite. High deformation temperatures promote dynamic recovery and dynamic recrystallization growth.
3. Dynamic softening was dominated by the DRV mechanism during hot deformation. Increasing temperature and decreasing strain rate promoted the DRX mechanism. Small particles (η, Al_3_Zr) can pin dislocations, promoting DRV by facilitating rearrangements and annihilating dislocations.
4. TiB_2_ and large second-phase particles generate high-density GNBs during hot deformation, serving as nucleation sites for DDRXed grains induced by the PSN effect. This enhances dynamic softening and contributes to improved formability. CDRX occurs in the vicinity of HAGBs induced by SIBM. The triple junction exhibits a stronger SIBM effect than that of Line-HAGB due to the more pronounced local stress.

## Figures and Tables

**Table 1:** Elemental composition of the TiB_2_/Al-Zn-Mg-Cu composite (wt.%).

Elements	TiB_2_	Zn	Mg	Cu	Zr	Al
Nominal component	6.0	10.0	2.3	1.6	0.12	Bal.
EDS results	4.5	10.3	2.1	1.8	3.96	Bal.

**Table 2:** Fitting parameters for the hot deformation behavior of the TiB_2_/Al-Zn-Mg-Cu-Zr composite.

*β*	*n*_1_	*α*	*n*
0.10	4.54	0.023	3.96

## Data Availability

Data are contained within the article.
